# Mediating religious disputes about children’s medical treatment: a qualitative study

**DOI:** 10.1136/bmjpo-2025-003789

**Published:** 2025-12-07

**Authors:** Jaime Lindsey

**Affiliations:** 1 University of Oxford, Oxford, UK

**Keywords:** Ethics, Child, Palliative Care, Qualitative research, Intensive Care Units, Neonatal

## Abstract

**Background:**

Mediation is a process which allows conflicting parties to come together with the support of a mediator to try and find an agreed way forward. Mediation has been increasingly used in paediatric medical treatment disputes, but there is little empirical data exploring participant experiences of mediation and/or the role of religion in resolving these disputes.

**Method:**

This qualitative study aimed to improve understanding of the role of mediation in resolving paediatric medical treatment disputes, in light of high-profile conflict about children’s medical treatment reaching the courts in England and Wales in recent years. Analysis of 17 semi-structured interviews with healthcare professionals, mediators and a family member of a patient was carried out and analysed thematically. The role of religion was an inductive theme identified in data analysis.

**Results:**

The analysis found that religion was a relevant factor for the majority of participants. Three themes in relation to the role of religion are identified: religious beliefs as a factor in the cause of conflict and entrenched views, religious beliefs and compromise in mediation, the role of religious support for family members in mediation. The findings show that there was no evidence to suggest that when religious disputes were mediated, it led to agreements undermining the child’s best interests, that the presence of religious views among family members did not mean that the dispute could not be mediated and that there may be benefit in having religious supporters present at mediation. Instead, the research showed that openness to mediation and openness to resolution was key.

**Conclusions:**

That mediation can be used in paediatric best interests disputes with a religious dimension and that mediators should develop further ways of delineating which cases could be effectively mediated. Finally, the article sets out some suggestions for areas of further research.

**WHAT IS ALREADY KNOWN ON THIS TOPIC**

- There is a lack of evidence regarding mediation’s role in resolving medical treatment disputes. Existing studies draw from case law analysis or discussion based on mediation’s use elsewhere. The small number of studies that do exist in England and Wales do not include direct participant experiences of mediation.

**WHAT THIS STUDY ADDS**

- One of the first empirical studies conducted with participants who have experience of mediation in paediatric medical treatment disputes in England and Wales. As a result of this study, we know that paediatric medical treatment disputes may still benefit from the use of mediation. This study adds evidence to support the view that mediation can still be considered in best interests disputes concerning children where family members hold religious beliefs, although mediators should carefully identify appropriate cases where the parties are open to mediation and resolution.

**HOW THIS STUDY MIGHT AFFECT RESEARCH, PRACTICE OR POLICY**

- The implications of this study are that mediation may be used in paediatric medical treatment disputes even where there is a religious dimension to the dispute. However, further analysis is needed to determine which types of cases are well suited to mediation, how healthcare professionals respond to strong faith-based reasoning and what role religion plays in conflict in paediatric disputes more widely.

## Introduction

 This article considers the role of mediation in helping to resolve conflict between healthcare professionals (HCPs) and family members in disputes about a child’s medical treatment. Mediation is a process which allows conflicting parties to come together with the support of an independent third party, a mediator, with the aim of trying to find an agreed way forward. Mediation is a flexible process, which can be tailored to individual needs. It will usually involve one or several meetings between the mediator and those in dispute. Mediation does not aim to resolve all aspects of conflict between HCPs and family members; rather, it allows people to come together in a neutral, carefully managed process, with the possibility of agreement on some aspects of the dispute.^1 2^ Mediation’s success ought not to be measured in terms of losing or winning; rather, as whether parties can benefit from the more therapeutic process that mediation may provide, including improved working relationships, enhanced communication and a safe place in which parties can feel heard and have their experiences acknowledged.^1^,^4^ This is an important distinction between healthcare mediation and mediation in other areas where notions of compromise and negotiated settlement have been more prevalent.^5^

Mediation has been increasingly used in paediatric medical treatment disputes about the child’s best interests, although mediation will often engage in wider aspects of the dispute going beyond best interests and exploring the underlying conflict. Best interests is a legal concept which protects the welfare of the child under the Children Act 1989. It requires any decision taken in relation to a child to be in their best interests.^6^,^8^ The concept is not limited to clinical interests and includes wider factors including the child’s own wishes and feelings, the risk of harm and personal factors such as age, sex and background. Those with parental responsibility under s2 Children Act 1989, usually but not always the biological parents, must also act in their child’s best interests when making medical decisions for them. However, where an HCP believes that the parents may not be acting in the child’s best interests, they can make an application to the High Court of England and Wales for a determination of what treatment (or withholding of treatment) is in the child’s best interests. Several of these disputes have been extremely high-profile in recent years, something which is not often seen in other jurisdictions.^6^ Arguably as a result of this high-profile conflict, mediation has been suggested by some judges as a better way forward for resolving best interests disputes. For example, Mr Justice Francis in the Charlie Gard case explained:

I recognise, of course, that negotiating issues such as the life or death of a child seems impossible and often will be. However, it is my clear view that mediation should be attempted in all cases such as this one even if all that it does is achieve a greater understanding by the parties of each other’s positions.^9^

However, recent case law has been more mixed, suggesting that mediation should be considered but not where it would cause delay which undermines the child’s best interests.^10 11^ Mediation of medical treatment disputes has not been widely researched and there is no data collected on mediation’s use, meaning we do not know the total number of mediations which take place.^10 12 13^ There has been some discussion of mediation’s role in disputes with a religious element, and mediation has been considered more widely as a way of resolving conflict.^3^,^512 14 15^ The role of chaplains in mediating conflicts over patient care has also been explored in the Polish context, with some evidence to suggest that this religious support can be beneficial.^16^ Building on this literature and the limited evidence base regarding mediation, this research is one of the first empirical studies to explore mediation in this field.^1 12^ The study is qualitative in nature and therefore does not seek to make claims that the findings are generalisable. It has been argued elsewhere that mediation for disputes about children’s medical treatment is unlikely to prevent litigation where there is a religious element or high conflict, and this paper responds to that argument to provide an alternative account of mediation’s potential based on in-depth interviews with people with experience of medical treatment disputes and mediation.^12^ This paper takes a different interpretation to argue that even disputes with a religious element may be amenable to mediation. Moreover, there are benefits to allowing family members who hold religious beliefs to have religious support within the mediation process. When referring to a ‘religious dispute’ here, I mean a disagreement about the child’s best interests where the family members of the child expressly held religious beliefs. This does not mean that the dispute was entirely centred on religious issues. In fact, they may only have been on the periphery of the dispute, but the role of religion was a prominent theme and therefore warrants specific analysis in this paper.

## Methods

This qualitative study in England and Wales looked at the use of mediation to resolve disagreements between HCPs, family members and patients about the provision of health and care to the adult or child patient. The primary research includes 30 semi-structured interviews with participants in mediation and/or professionals with experience of medical treatment disputes, conducted by the author or members of the research team. 17 interview participants had experience of children’s medical treatment disputes, and 18 participants had experience of adult health and care disputes. This article focuses on the evidence from children’s disputes only, although some themes were identified across the data. Inclusion criteria for the interviews were:

1. Have either taken part directly through attendance at a medical treatment mediation in England and Wales since 31 December 2012, or have professional experience of medical treatment disputes;
2. Have taken part in the above in capacity as either (1) a HCP, (2) a patient, (3) a patient’s supporter, family or friend, (4) a mediator and (5) a legal professional.

Exclusion criteria were:

1. An adult patient who lacks the capacity to take part in the interview research.
2. A child patient who is not Gillick competent to take part in the interview research.
3. A child patient who is going to turn 16 during the study.

Participants were recruited through purposive and snowball sampling, through email contact with mediators and other professionals who shared our request with mediation participants who met our inclusion criteria. Several potential participants were contacted directly and the project was listed in the National Institute for Health Research Portal. Empirical data collection commenced in February 2023 and was complete by January 2025.

Interviews were conducted online (n=16) and in person (n=1), depending on interviewee preference. Interviews followed a semi-structured format and followed an interview schedule. Informed consent was obtained from participants and all names used in this research are pseudonyms. Interviews were audio recorded with consent of the participants and uploaded to a secure server for transcription. These were then reviewed and anonymised, saved to the secure university server and uploaded for analysis to NVivo.

Of the 17 interview participants with experience of children’s medical treatment disputes, the majority were HCPs (n=7), followed by mediators (n=3), lawyers (n=3), chaplains (n=2) and family members (n=1) or family supporters (n=1). Three of these 17 participants did not have direct experience of taking part in a mediation but had professional experience of medical treatment disputes or mediation within their department (see table 1 for further demographic information which also sets out the experience of individual participants). The minimum interview length was 30 min and the maximum was 100 min. Interviews with HCPs and mediators were deemed complete when data saturation was reached. Due to the challenges in recruiting family members, data saturation was not reached for this group. Difficulties in engaging with family members arose because mediation is a confidential process and, therefore, recruitment relied on mediators identifying possible participants and sharing the request. However, the sensitive nature of the subject matter and timing of any request (ie, if immediately following a mediation) raised challenges in respect of family members’ willingness to revisit the issue.

**Table 1:** Interview participants with experience of children’s medical treatment disputes

Pseudonym	Primary role	Secondary role	Sex	Direct experience of mediation	Level of experience
Ed*	Mediator	Solicitor	M	Y	More than 20 years’ experience in litigation and mediation
Elizabeth†	Mediator	None	F	Y	More than 5 years’ experience of mediation
Abigail‡	Mediator	HCP	F	Y	More than 5 years’ experience of mediation and more than 10 years’ experience as HCP
Rowan‡	HCP (paediatric intensive care consultant)	None	M	Y	More than 10 years’ experience as HCP
Francesca‡	Chaplain	None	F	Y	More than 10 years’ experience as hospital chaplain
Jack‡	HCP (paediatric intensive care consultant)	None	M	Y	More than 10 years’ experience as HCP
Marcus‡	Chaplain	None	M	Y	More than 20 years’ experience as hospital chaplain
Yasmin‡	HCP (consultant paediatrician)	Mediator	F	N	More than 10 years’ experience as HCP
Rachel‡	Lawyer (children’s cases)	None	F	N	More than 10 years’ experience as lawyer
Kai‡	HCP (neonatologist)	None	M	Y	More than 20 years’ experience as HCP
Lola‡	Family (mother)	N/A	F	Y	More than 10 years’ experience as parent and trained nurse
Tamara	HCP (consultant paediatrician)	None	F	Y	More than 10 years’ experience as HCP
Laura‡	Family supporter	Lawyer	F	Y	More than 20 years’ experience supporting families
Nadine	Lawyer (adult and children’s cases)	Mediator	F	Y	More than 10 years’ experience as barrister and more than 5 years’ experience as mediator
Sonny*	HCP (consultant paediatrician)	None	M	N	More than 10 years’ experience as HCP
Oscar*	Lawyer (adult and children’s cases)	None	M	Y	Over 20 years’ experience as lawyer
Caleb‡	HCP (paediatric intensive care consultant)	None	M	Y	Over 20 years’ experience as HCP

* Three interviews where religion was raised as an issue.

† Two participants were interviewed twice. The first because she was subsequently involved in an observed mediation and we wanted to follow up specifically on that experience. The second, because he had more to say about his experience of mediation than the initial interview permitted, so a further interview was arranged.

‡ 12 interviews where religion was a key theme.

HCP, healthcare professional; N/A, not available.

Data were analysed using a combination of thematic analysis and a therapeutic justice theoretical framework.^17 18^ Data analysis was conducted by two researchers (Gillian Francis and JL) initially through familiarisation with the data, followed by the identification of codes which set out the key emerging themes. Once codes were agreed on, subcodes were created which provided more granular analysis of the emerging themes. These subcodes were checked against the data and the final stage of data analysis was interpretation and finalisation of the findings. Here, the focus is on one emerging theme from the data, specifically the role of religion in mediation of children’s medical treatment disputes. This theme was identified inductively and was not part of the deductive theoretical framing of the research project.

### Patient and public involvement

There was no patient involvement in this research due to the nature of the study focusing on medical treatment disputes where most patients are very unwell or the subject matter related to end-of-life care. This was anticipated in advance of the start of the study. This meant that young people were not directly involved, which is a limitation of the research. However, family members of patients were involved in the study, both as participants in the research and in relation to study governance, for example, a family member attended our research advisory group meeting. Results of the research will be shared with all participants where consent was provided for us to do so.

## Results

Religion appeared as a factor in three ways: its role in the cause of conflict and entrenched views, religion and compromise in mediation, the role of religious support for family members in mediation. These themes, which emerged from inductive analysis of the interview data, show that religion was a relevant factor for the majority of participants in the research even where not explicitly asked about this. Indicative quotes from the interviews are provided in the boxes below and participant data is included in table 1.

### Religion, entrenchment and causes of disagreement

Religion was raised in several cases as a contributory factor to the initial breakdown in relationships between HCPs and family members in best interests disputes. There were several concerns about how family members with religious views were responded to by HCPs. Evidence of this is shown in box 1 and our interview with Lola (patient’s mother) is indicative of this. Lola’s dispute with the HCPs concerned her son who was born with a rare mitochondrial disease and, within 5 days of his birth, suffered from breathing difficulties and was put on life support treatment. Because of the low survival rate for his condition, the clinical team had suggested that life support be withdrawn, but the child’s parents did not agree to this and he remained in hospital. In this case, Lola’s religion and faith were important to her and she would often cite the position ‘let God be God’ in relation to making a decision about her son’s care. Lola also told us that she felt as if these religious views were weaponised against her by the treating team. Yet Lola further explained that the issue for her was not to do with her religion or that she held an entrenched position because of her religion. In fact, she ultimately did agree to the withdrawal of treatment following an independent mediation process. Lola felt that her religious beliefs were not the barrier to her acceptance of the proposed treatment, nor did she think she was acting contrary to her child’s best interests.

Box 1 **Quotes linked to religion, entrenchment and causes of disagreement**

‘[the parents] felt that their religious views were being used against them, and they were being regarded as almost a sign of mental health… they were feeling that [their views] were being weaponised against them.’ (Abigail, mediator)

‘And then they went as far as writing to ethics, and in one of the places the ethics asked, “Okay, what was the challenge?” They said… what was the—“Any mental health challenge?” They put under the mental health, “The mother believes in God.”’ (Lola, patient’s mother)

‘I was slightly dubious at that point as to whether it [mediation] would be of any benefit, because this family’s views were … very clearly based around their religious views and the sanctity of any life.’ (Jack, paediatric intensive care consultant)

‘I think it’s probably wrong to say that because somebody’s got very strong faith-based views they are unlikely to change their mind… Because I think within all faith communities there are a range of viewpoints’ (Marcus, chaplain)

‘It’s not if you’re pro-life that you’re going to—that you become somebody that can’t think rationally or sensibly or calmly about proper outcomes.’ (Laura, Family supporter)

‘She's [patient’s mother] a—Christian, is her background. There’s … very often … a faith cause for these conflicts … And you know, she’s absolutely convinced in, that the child responds to her and to various stimuli in the environment which nobody else can tell and that God is going to make the child better. And she is a very aggressive woman and she shouts and likes to intimidate people whenever there's a meeting and unfortunately the mediation was no different.’ (Caleb, paediatric intensive care consultant)

The research also found that there was an overlap between parties having entrenched views in mediation and there being a religious element to the dispute. On the face of it, this might suggest that cases with religious family members involved are not well suited to mediation. However, we found that it was not the religious views per se that obstructed resolution through mediation. Rather, it was the attitude of the parties involved that was the most relevant factor in making mediation an effective process, for example, the extent of their hostility towards the HCPs. Several HCPs, two chaplains and one family member suggested that there was complexity among the religious views of families and from their experience religious family members were able to change their minds on the issue before them at mediation, even where they initially appeared entrenched in their positions due to religious beliefs. The inference from these discussions was that the mere presence of religious views among family members does not mean that the dispute cannot be mediated, although this may be the perception that some people hold because mediation is (wrongly) seen as being about compromise.^19 20^ Instead, the focus should be on selecting suitable cases for mediation, including considering the attitude of the parties and their openness to mediation, rather than the focus on religious beliefs, something explored further in the discussion section below.

### Mediating religious disputes and compromise over best interests

Considering these concerns in the context of mediation, we did not find any evidence to suggest that religious disputes specifically, when mediated, led to an agreement undermining the child’s best interests (see box 2 below).^20^ In most cases, family members with religious beliefs either accepted the recommendations of the treating team, and agreements to withdraw treatment were seen in four of the interviews (Kai, Lola, Abigail, Marcus) or declined to accept the recommendations and proceeded to court hearing. Contrary to a perception among some that religious beliefs can ‘stonewall a secular approach’^15^ there was no evidence to suggest that mediation was used by family members to pressure HCPs into changing their mind and agreeing with their religious perspective. We identified one clear example (interview with Caleb, HCP) where the child’s best interests may have been undermined in a religious dispute that was mediated. But it was not the religious aspect itself which caused the child’s best interests to be undermined, if at all; rather, it was the delay in reaching resolution (at least 18 months) which could be argued as negatively impacting on the child’s best interests.^6 7 20^ There were several contributory factors to this, including delays in getting a second opinion, attempts to resolve the matter internally, and then, finally, mediation. In contrast to this one example of delay, other positive outcomes were achieved through mediation in religious disputes; specifically, these included agreements about the child’s care that would be tested and then returned for discussion in the following mediation meetings, agreements to trial an alternative medication, agreements to allow prayer services at the hospital and agreements to withdraw life-sustaining treatment.

Box 2 **Quotes linked to religion and compromise**

‘the mediation process is on to a loser there from the outset’ because ‘the clinical teams aren’t left with anything to give’(Caleb, paediatric intensive care consultant)

‘was it [the agreement reached at mediation] in the child’s best interests? Yes, I think it was. And the indication of that I think is that both parties were settled in it. Even the parents who didn’t, who wanted the child to be … given another six months. But sort of the decision was made after that month that it would extubate and I think that’s something they could settle on’ (Abigail, mediator)

‘I think where it [mediation] doesn’t work, is where there isn’t an option for a middle ground, where there isn’t a compromise option available … for example … do you put a child on a ventilator or you don't put them on a ventilator? And when you have that kind of a binary option, and there isn’t a half a ventilator you can have, then, then I'm not convinced that mediation has much role to … But if it’s a, a question of degree of, you know, do we, do we admit the child to intensive care, or just treat them on the ward, then, then you can see there’s a role for mediation in helping make those sorts of decisions, where there is room for movement on either side.’ (Jack, paediatric intensive care consultant)

If … there’s a binary decision and it just needs a decision made and … the, the two parties are, for religious or moral or ethical views, completely opposing, I don't think there's any role for mediation. Because in order to change your views on that point you gotta change someone’s morals or ethics, and then that, that’s wrong…’ (Jack, paediatric intensive care consultant)

### The role of religious support for families in mediation

We found evidence that participants felt that mediated disputes with a religious element would benefit from religious supporters being part of the mediation process. As indicated in box 3, our data suggest that ministers of religion can: support family members emotionally and spiritually before, during and after the mediation; provide advice and clarification on religious doctrines; and provide spiritual ways forward which can be made part of the agreements reached at mediation. The positive role religious support might play was particularly notable in the interviews with chaplains, the family member and the family supporter. An important distinction was made by some participants, however, between religious support for family members with genuine faith-based concerns and the presence of religious groups where such groups may have an interest in promoting a wider agenda beyond the support for the individuals in the specific case being mediated.

Box 3 **Quotes links to religious support**

sometimes people have very strong viewpoints, but when they’re faced with a particular situation they see things slightly differently. And within all faith communities, there is different viewpoints, so it may be that somebody might be coming from a particular viewpoint, but haven’t heard another point from their tradition or from within their faith community. And actually helping them understand that there is a range of viewpoints early enough stops that blinkering and banging together of heads so it would be about and again.’ (Marcus, chaplain)

‘I think they had their, you know, local religious leader of the church, and whom they were in constant discussions with. That particular person necessarily—didn’t necessarily directly interact with us. I think they had their local—they had that support. But the hospital chaplain was there in all the mediation meetings. They were sat there. They sometimes didn’t have anything to say, but they were there almost, like, as a support for the family.’ (Kai, neonatologist)

‘the family have got themselves stuck in a place based on a faith claim that’s not quite right, and our own chaplains are perceived to be a bit of the hospital and then you get someone in from their life and that can be super helpful.’ (Rowan, paediatric intensive care consultant)

‘the Muslim chaplain in the hospital is able to come and talk to, and we sit in on those meetings often, and … they’re able to change the family’s opinion because … he speaks their language essentially.’ (Caleb, paediatric intensive care consultant)

## Discussion

This research did not find any evidence to suggest that disputes where parties held an expressed religious belief, when mediated, led to agreements which undermine the child’s best interests. In all cases, participants with religious beliefs either fully or mostly accepted the recommendations of the treating team following mediation (eg, they agreed with the proposed outcome with only minor variations such as location of withdrawal or agreement to allow prayers before withdrawal of treatment), or they declined to accept the recommendations and the dispute continued and, in most cases, proceeded to litigation. There were no examples provided where religious family members used mediation to obtain an agreement which explicitly undermined the child’s best interests, either subjectively as described to us from the opinion of the HCP or mediator, or objectively from a legalistic understanding of the facts knowing how courts are likely to decide these matters.^5 10 20^ This is an important observation because there is concern, particularly among lawyers and the judiciary, that mediation can lead to agreements which undermine the child’s best interests, which we did not find evidence for.^1 18^ The only example identified where the child’s best interests were potentially undermined in a mediation with a religious element was in Caleb’s example, discussed above. However, our interpretation of the issue in that case was the delay to resolution which may have undermined the child’s best interests rather than the religious compromise in mediation.^20^

The religious dimensions to these disputes were often more nuanced than the widely held view in the literature suggests.^5 12^ The data indicate that those with religious viewpoints are able to change their position when presented with evidence or where able to improve their understanding of the situation that faces their child. That is not to suggest that mediation will always be appropriate. As several participants explained, it may feel easy to take a blanket response to religious objections, but actually, it will depend on the facts of the case and the attitude of the parties as to whether mediation is appropriate.

This research suggests that it is not religion itself that is the barrier to effective mediation, but that other factors may prevent the parties from being able to collaborate to achieve a resolution or, at the least, improved understanding of the other.^17^ As noted above, the attitude of the parties and their openness were important factors in the dispute continuing or not resolving at mediation. Parties should be open to engaging in the mediation and open to reaching resolution. This openness will include being willing and able to follow the mediation process, ground rules and agenda that is agreed with the mediator, which is important to ensure the parties are taking part in the mediation voluntarily and do not feel coerced or pressured into doing so.^17^ Openness to resolution was a key factor in reducing conflict. This was clear from several mediator participants who explained there are cases they will not mediate:

Because there is one I think I would refuse… It’s something where with the person says ‘I only want this and nothing’s gonna change my mind’, then I might say ‘I’m not… I don’t think I’m not sure there is territory for mediation’. So, I’ve had situations like that and uhm although of course generally people come to mediation saying ‘I only want this’, so again I try to distinguish between. (Abigail, mediator).

If one or both parties are completely closed in their approach to resolution, then the conflict can become entrenched and difficult to resolve, irrespective of whether this is underpinned by a religious belief. Relatedly, if the parties have a pre-existing relationship characterised by severe hostility or threats, then this conflict can be difficult to resolve, even with mediation. One participant (Nadine, lawyer) suggested that there are some cases where the family, for example, may have been ‘really unpleasant to the doctors and kind of accused them of being murderers and … behaved really badly towards them’, and those cases may be ‘too late to try and salvage’. In those cases, the family members may or may not hold religious beliefs, but it is not the religious beliefs themselves that cause hostility as many religious parents do not react in this way, as exemplified by Lola’s case. Mediators should develop mechanisms for delineating between cases with a religious element which could be effectively mediated because the parties are open to mediation and resolution, and those which are less likely to be suitable because the parties are too hostile towards each other and closed to resolution.

In conclusion, participants gave repeated examples of the benefits of mediation where family members held religious beliefs, particularly where parents could draw on their religious support networks for assistance within the mediation process. HCPs may feel resistant to including this support in mediation, and it is for the mediator to set ground rules regarding the participation of different parties to make the mediation a participatory and open process for all.^1321^,^23^ Finally, it would be worthwhile conducting further research beyond that which is already published exploring the prevalence and role of religious views among parents in children’s healthcare settings generally, compared with the prevalence of religious views where disputes escalate to conflict and, ultimately, remain unresolved before mediation or litigation,^15^ as well as research to consider the impact of mediators’ own religious beliefs in these disputes.

## Data Availability

No data are available.
