# Supplementary material for: Mediating religious disputes about children’s medical treatment: a qualitative study
Source: BMJ Paediatr Open. 2025 Dec 7;9(1):e003789. doi: 10.1136/bmjpo-2025-003789 (PMC12684158; doi:10.1136/bmjpo-2025-003789)
Supplement: Supplementary file 1 [file bmjpo-2025-003789.reviewer_comments.pdf]

PEER REVIEW HISTORY

BMJ Paediatrics Open publishes all reviews undertaken for accepted manuscripts. Reviewers are asked to complete a checklist review form and are provided with free text boxes to elaborate on their assessment. These free text comments are reproduced below.

ARTICLE DETAILS

**TITLE (PROVISIONAL)**	Mediating religious disputes about children's medical treatment: a qualitative study
**AUTHORS**	Lindsey, Jaime

VERSION 1 - REVIEW

**REVIEWER NAME**	Ana Milena Antolinez Portilla
**REVIEWER AFFILIATION**	Hospital Universitario Arnau de Vilanova, Uffis Cuidado paliativo
**REVIEWER CONFLICT OF INTEREST**	N/A
**AI DISCLOSURE**	No
**DATE REVIEW RETURNED**	18-Jul-2025

**GENERAL COMMENTS**	This work provides interesting data for a debate that takes place daily in hospital settings, especially in those where invasive procedures are commonplace, such as intensive care units. Given the very limited body of evidence, this work is therefore relevant.<br>A qualitative approach is appropriate for this type of question, and while the recruitment methodology may increase the risk of bias, the small sample size makes it necessary.<br>I believe that the methodology section should include more information on the inclusion and exclusion criteria.<br>The same section mentions that 17 of the participants have experience in disputes over medical treatments in pediatrics. It would be helpful for readers to understand what was considered experience, since the information provided in Table 1 is limited to reporting a number of years, and for some participants, these years refer to their professional practice. If it is not possible to clarify this, I suggest limiting the information to stating that they were the mediators in the cases in question, without emphasizing their level of experience, since this information is not essential given that the study does not seek to measure the outcome of the mediations per se.<br><br>The interview sections provide information; however, given their focus on the religious aspect, it would be interesting to evaluate whether there is a difference between religious and non-religious mediators in their perception of the problem, if this is possible.<br><br>In the discussion section, when referring to the outcomes, it is unclear whether mediation achieves its objective in most cases or not. I suggest revising the wording (lines 27-32).<br><br>Finally, the last paragraph of the discussion addresses problems that have been recognized as barriers to reaching consensus with families in advanced life support settings, and I believe that the proposal to expand the research to less critical settings is relevant.

**REVIEWER NAME**	Diego Fernando Puerto Torres
**REVIEWER AFFILIATION**	Universidad Nacional de Colombia
**REVIEWER CONFLICT OF INTEREST**	N/A
**AI DISCLOSURE**	No
**DATE REVIEW RETURNED**	07-Aug-2025

**GENERAL COMMENTS**	Congraulations for the use of qualitative methodologies to investigate such specific and poorly documented relationships. This research can be a very important guideline for understanding the importance of mediation in promoting and protecting the patient interests<br>Some possible editorial corrections: Do not use the same word consecutively. For example: "in the research. The research”" (results, p. 1).<br>Conceptual clarifications: I consider it necessary to have a reference definition for the most important technical terms: "religious dispute" or "best interests." For example, greater precision is needed regarding what a religious dispute is: (differences over whether or not to belong to a religion, differences in the practice of worship, values, conceptions of life, etc.) This is something that can probably be gleaned from the research data.<br>Although all the methodological requirements are sufficiently described, no mention is made of the participation (or lack thereof) of children in the research. Nor is it clarified whether the research assumes that children are "represented" by their parents. If the “best interests” of children are essential to the research, it is necessary to clarify whether children participated in the research. It may be that they were not included, in which case this should be recognized as a shortcoming of the research and a challenge for future research.<br>Again, considering the methods and results, it would be helpful to specify the concept of “best interest.” Although it may be clear to familiar readers, a basic definition of the concepts surrounding the disputes is necessary.<br>The article should delve into the findings and strengths of the research. For example, delve into the issue that “it was the attitude of the parties involved that was the most relevant factor in mediating” rather than the presence of religious beliefs per se. If it is found that the key factor is people's attitudes toward mediation, a more detailed description is necessary. Furthermore, what “there was greater openness” means should be specified, as expressed by the interviewees.<br>The descriptions in sections 2 (Mediating religious disputes and compromise over best interests) and 3 The role of religious support for families in mediation could be more detailed. For example, by describing the ways in which best interests could be a driving force for the advancement or stagnation of mediations, or situations where the choices are binary, as in the excerpt from Jack (pages 11-12).<br>The contrasts allowed by the collected data can be addressed in more detail. It is advisable to specify what you want to exemplify with each interview excerpt. In some cases, it is not clear what you want to show with a excerpt.<br>More emphasis should be placed on the conclusions that the research does allow; for example, I recommend a more detailed description of the importance of the attitudes of the parties involved in the mediation. Additionally, since it is mentioned in the discussion, it is advisable to include an analysis of healthcare professionals' resistance to participating in mediations.

VERSION 1 – AUTHOR RESPONSE

Response to reviewers

I am grateful to the two reviewers for their constructive and supportive peer review comments which have helped me to improve the article significantly. I have accepted all of the suggestions as set out below and changes to the article can be seen in track. I also attach a clean version of the article where changes have been accepted.

1. Patient and Public Involvement:

Authors must include a statement in the methods section of the manuscript under the sub-heading 'Patient and Public Involvement'.

This has been amended in this methods section.

Rationale & Required Changes

1. Conceptual Definitions (Reviewer 1 – Major)

• “Religious dispute” and “best interests” must be clearly defined upfront, with citation of a standard source or a brief conceptual paragraph in Methods.

This has been amended in the introduction with definitions for each of these concepts.

• Clarify the role (or non-role) of children in the research: explicitly state whether children themselves were interviewed or merely represented by parents, and acknowledge this as a limitation if applicable.

This has been amended in the methods section.

2. Depth of Results (Reviewer 1 – Major)

• Expand Sections 2 and 3 to explore in detail:

• How “attitude of parties” emerged as the key mediator of success, beyond mere presence of faith.

This factor has been expanded in the final discussion section to show the other causes of conflict or challenges for mediating religious disputes.

• What “greater openness” entailed in practice, using selected illustrative excerpts.

As above, this factor has been expanded in the final section.

• Specific examples of compromise pathways (e.g. trial of alternative medication, staged withdrawal).

More detail on this has been added at p11-12.

3. Presentation & Editorial Corrections

• Expand and clarify the Abstract: flesh out key objectives and findings.

• Enhance clarity and flow throughout by using precise, varied word choice and correct grammar.

• Eliminate repetitive phrasing (e.g., “in the research. The research”).

• Prune less-informative quotes, retaining only those that meaningfully illustrate key themes.

This has been amended throughout the revised draft and the abstract has been developed in more detail.

4. Methodological Clarity (Reviewer 2 – Minor)

• In Methods, provide fuller detail on inclusion/exclusion criteria and clarify what constituted “experience” for the 17 participants (years in role vs. direct mediation involvement).

• In Discussion, refine language on outcomes: state explicitly how often mediation achieved settlement vs. proceeded to litigation, avoiding ambiguity over whether “majority” succeeded.

More detail on exclusion/inclusion criteria has been added to the method section. I have tried to use more precise language about outcomes but this is sometimes difficult as the research is qualitative and so this information was not always available.

5. Exploratory Suggestion (Reviewer 2)

• Where feasible, comment briefly on any observed differences between religious vs. non-religious mediators in perceiving or managing disputes; if data are insufficient, note this as an area for future research.

This was not noted as part of the research but has been added as a possibility for further research in the final section.

VERSION 2 - REVIEW

**REVIEWER NAME**	Ana Milena Antolinez Portilla
**REVIEWER AFFILIATION**	Hospital Universitario Arnau de Vilanova, Uffis Cuidado paliativo
**REVIEWER CONFLICT OF INTEREST**	N/A
**AI DISCLOSURE**	No
**DATE REVIEW RETURNED**	19-Aug-2025

**GENERAL COMMENTS**	I believe the article is much clearer after the modifications made based on the recommendations made by all the reviewers.

**REVIEWER NAME**	Diego Fernando Puerto Torres
**REVIEWER AFFILIATION**	Universidad Nacional de Colombia
**REVIEWER CONFLICT OF INTEREST**	N/A
**AI DISCLOSURE**	No
**DATE REVIEW RETURNED**	22-Aug-2025

**GENERAL COMMENTS**	Thanks to the author for accepting the suggestions. I think the article has improved, especially in the methodological section. One last suggestion for future articles: although including several interview excerpts anchored in an analysis is positive, I think it's a better option to explain the contributions of each excerpt individually and then provide a general analysis.
